# The clinical potential of gene editing as a tool to engineer cell-based therapeutics

**DOI:** 10.1186/s40169-020-0268-z

**Published:** 2020-02-07

**Authors:** Candice Ashmore-Harris, Gilbert O. Fruhwirth

**Affiliations:** 1 Imaging Therapy and Cancer Group, Dept of Imaging Chemistry & Biology, School of Biomedical Engineering & Imaging Sciences, St Thomas’ Hospital, King’s College London (KCL), London, SE1 7EH UK; 2 Centre for Stem Cells & Regenerative Medicine, School of Basic and Medical Biosciences, Guy’s Hospital, KCL, London, SE1 9RT UK

**Keywords:** Cell therapy, Clinical trial, CRISPR–Cas9, HIV, Oncology, TALEN, Zinc finger nuclease

## Abstract

The clinical application of ex vivo gene edited cell therapies first began a decade ago with zinc finger nuclease editing of autologous CD4^+^ T-cells. Editing aimed to disrupt expression of the human immunodeficiency virus co-receptor gene *CCR5*, with the goal of yielding cells resistant to viral entry, prior to re-infusion into the patient. Since then the field has substantially evolved with the arrival of the new editing technologies transcription activator-like effector nucleases (TALENs) and clustered regularly interspaced short palindromic repeats (CRISPR), and the potential benefits of gene editing in the arenas of immuno-oncology and blood disorders were quickly recognised. As the breadth of cell therapies available clinically continues to rise there is growing interest in allogeneic and off-the-shelf approaches and multiplex editing strategies are increasingly employed. We review here the latest clinical trials utilising these editing technologies and consider the applications on the horizon.

## Background

Cell therapy is defined as the administration of live cells to a patient with the aim of repairing or replacing damaged cells or tissues. It relies on a predefined cell population, which can either be from the same patient (autologous) or from a different donor (allogeneic). The type of therapeutic cell varies widely with clinical trials currently dominated by haematopoietic cells, mesenchymal signalling cells [1] and lymphocytes, but also, at a lower frequency, dendritic cells, hepatocytes, epithelial cells and various others are being investigated [2, 3]. From a disease perspective, oncology is responsible for over half of all cell therapy trials [2]. Notably, the emerging arena of cell therapies has been boosted by several approvals in recent years [4]. Unlike other treatments, cell therapies are live cell products and, via genetic engineering, can be enhanced to achieve better efficacy or tailored to individual patients. Importantly, they require extensive characterisation to demonstrate safety and compatibility. In this context it is noteworthy that their in vivo distribution, survival and efficacy at target, but also off-target tissues are critical parameters. For example, off-target activities led to severe adverse effects, with other life-threatening side effects and fatalities during clinical trials also reported. This together with the fact that most clinical cell therapy trials are still performed without knowledge about the in vivo distribution and fate of the administered therapeutic cells resulted in suggestions to routinely implement in vivo cell tracking (by imaging) [5–7] and suicide genes [8]. As better efficacy and tailoring to patients is increasingly achieved through genetic engineering, the latter safety-related aspects can be piggy-backed into therapeutic cells at this stage [9]. Traditionally, genetic engineering has been achieved through the use of viral vectors (e.g. γ-retroviruses, lentiviruses), which more or less randomly integrate the transgenes into the genome [10]. This approach is often also classified as ‘gene therapy’ and has been applied for cell therapies in diverse aetiologies, for example, ranging from cancer immunotherapies to regulation of immune tolerance in autoimmune diseases [11]. A specific form of genetic engineering is gene editing, which offers a much more specific way of integrating a desired genetic payload at a distinct location into the genome of target cells [12]. As the breadth of cell therapies available clinically continues to rise and gene editing approaches provide potentially game-changing opportunities, we review here the latest clinical trials employing this new technology.

## The narrow-winding road to genetically engineered cell therapies

Whilst genetic engineering strategies have generally advanced cell therapies to great patient benefit, the journey has not been smooth. The first report of successful cell therapy engineering was in X-linked severe combined immunodeficiency (SCID-X1) patients and involved collecting patient CD34^+^ hSCs, transducing them ex vivo with a replication-deficient γC Moloney retrovirus containing the γC cytokine receptor common subunit gene, an X-chromosome linked gene that is inactivated in SCID-X1 patients rendering them devoid of mature T and Natural Killer (NK) cells [13, 14]. The goal of the approach was to restore patient capacity to form mature T and NK cells. The engineered hSCs were reinfused into patients and within 10 months positive results were seen, with patient T and NK compartments filled by γC transgene-expressing cells. Unfortunately, nearly 3 years post-infusion two patients developed leukaemia as a result of clonal expansion of engineered T-cells. Both patients had proviral insertions which led to activation of the proto-oncogene *LMO2* causing exponential proliferation of these cells [15]. Viral vectors had allowed both robust transgene expression and high engineering efficiency, but also caused the downstream disease and thereby rendered it clear that an improved understanding of the long-term risks of genetic engineering was required. In the years that followed this study scores of new vectors were designed to reduce the potential for insertional mutagenesis and improve safety [10], but some in the field were already looking at a more precise strategy of introducing transgenes at defined locations in the genome.

## Development of site-specific genetic engineering methods

Following the discovery that DNA double-strand breaks (DSB) could induce repair, scientists looked to exploit the repair process in order to manipulate cells with single base pair precision. Distinct nucleases with the capacity to recognise specific DNA sequences of interest (recognition sites) in endogenous mammalian genes were engineered, which could also cleave the DNA at these sites. Researchers were following the principles of homing endonucleases first discovered in budding yeast to do so [16], and laid the foundations of what became known as ‘gene editing’. These targeted editing approaches are now widely exploited in both preclinical and clinical research.

**Fig. 1:**
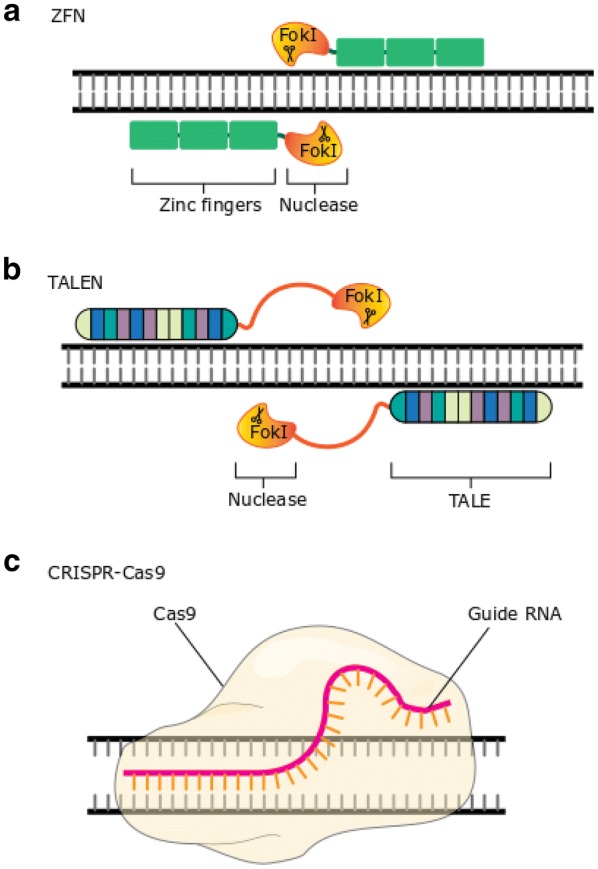
Gene editing technologies used in cell therapies. Depicted are the three basic structures and main characteristics of each editing platform used clinically in cell therapies showing how the editing agent interacts with the DNA in order to initiate the double-strand break. **a** Zinc-finger nucleases (ZFNs) consist of Zinc-finger proteins bound directly to an endonuclease such as FokI. The zinc finger proteins work as DNA-binding domains recognising trinucleotide DNA sequences, with proteins linked in series to enable recognition of longer DNA sequences, thereby generating sequence recognition specificity. The fused FokI functions as a dimer so ZFNs are engineered in pairs to recognise nucleotide sequences in close proximity ensuring DSBs are only produced when two ZFNs simultaneously bind to opposite strands of the DNA. **b** Transcription activator-like effector nucleases (TALENs) consist of bacterial TALE proteins fused to endonucleases such as FokI. As with ZFNs this requires paired binding to initiate the DNA break. Here the DNA targeting specificity comes from the modular TALE arrays which are linked together to recognize flanking DNA sequences, but each TALE recognises only a single nucleotide. **c** The CRISPR/Cas9 platform does not rely on protein-DNA binding as with ZFNs and TALENs but gets its DNA targeting specificity from Watson–Crick RNA–DNA base pairing of the guide RNA (gRNA) with the recognition site. Initially the Cas9 binds to a protospacer adjacent motif (PAM) this is a 2–6 base pair DNA sequence which is specific for each Cas protein. Without the correct PAM sequence the Cas will not bind or cut the DNA. Following correct PAM identification, the Cas melts the remaining target DNA to test sequence complementarity to the gRNA. PAM binding allows the Cas protein to rapidly screen potential targets and avoid melting lots of non-target sequences whilst searching for fully complementary sequences

Zinc-finger nucleases (ZFNs) were the first designer nucleases, produced from a naturally occurring transcription factor family known as zinc finger proteins, fused to FokI endonuclease. The zinc finger proteins work as DNA-binding domains recognising trinucleotide DNA sequences, with proteins linked in series to enable recognition of longer DNA sequences, thereby generating sequence recognition specificity. The fused FokI functions as a dimer [17], so ZFNs are engineered in pairs to recognise nucleotide sequences in close proximity (Fig. 1a). This ensures DSBs are only produced when two ZFNs simultaneously bind to opposite strands of the DNA, whereby the sequence recognition specificity is determined by the length of aligned DNA-binding domains. This limits off-target effects, but with the downside that arrays of zinc finger motifs influence neighbouring zinc finger specificity, making their design and selection challenging [18–20]. Early studies relied on delivery of the ZFN expression cassette to cells via DNA fragments derived from viral vectors. Studies later progressed to using mRNA delivery via electroporation to enable entry into target cells. This approach offers transient but high levels of the expression cassette within cells, presenting a lower risk of insertion/mutagenesis at off-target sites as a result of the shorter mRNA half-life compared to DNA [12]. This improved safety profile is paired with the benefit of highly efficient transfection (with levels > 90% reported) and excellent cell viability (up to 80%) [21–23].

Transcription activator-like effector nucleases (TALENs) were the next development following ZFNs. They also employ endonucleases such as FokI to initiate the DNA break, requiring paired binding, but the DNA targeting specificity comes from the fused bacterial TALE proteins [24, 25]. As with ZFNs, modular TALE arrays are linked to recognize flanking DNA sequences, but each TALE recognises only a single nucleotide and has no impact on the binding specificity of its neighbour, offering an improvement over ZFNs and a straightforward design process (Fig. 1b). As with ZFNs, for ex vivo cell therapy gene editing most TALEN-mediated approaches rely on mRNA as the delivery vector, with cell entry facilitated via electroporation.

The most recent system to be developed for gene editing is the clustered regularly interspaced short palindromic repeats (CRISPR) system. CRISPR originates from bacteria and uses a guide RNA (gRNA) which binds to the DNA target site. Subsequently, a nuclease, such as the CRISPR associated protein 9 (Cas9), induces conformational changes before cleaving the DNA (Fig. 1c). Targeting is accomplished through the gRNA molecule, which can be designed to optimise hybridisation with the sequence of interest. This can be done by standard Watson–Crick base pairing but must be followed by a DNA motif called a protospacer adjacent motif (PAM) to enable Cas binding. Like ZFNs and TALENs CRISPR–Cas9 gene editing can be achieved utilising electroporation of the Cas9 mRNA and gRNA but nuclease editing efficiency via this mode of delivery is limited due to instability of the unmodified sgRNA [26, 27]. Chemical stabilisation of the gRNA can limit its degradation, allowing time for the Cas9 protein to be translated following electroporation, and this improves editing efficiency [28]. Alternatively, a ribonucleoprotein (RNP) complex formed as a result of in vitro transcribed guide RNA incubation with Cas9 protein can be used for delivery. RNP complexes offer increased stability, higher editing efficiency and reduced cytotoxicity [26, 27]. They also benefit from accelerated nuclease kinetic activity, potentially reducing the activity window of the nuclease and thus opportunities for off-target effects [27–29]. Whilst some report that CRISPR can lead to increased DNA cleavage at off-target sites compared to the paired binding approaches of ZFNs and TALENs, strategies to reduce off-target activity are underway [30]. These include the quantification of imperfect Cas9-induced DSB repair products through primer-extension-mediated sequencing (PEM-seq) to improve determination of editing specificity and efficiency; as well as the use of high-fidelity Cas9 variants. Though there are distinct advantages and disadvantages for employing each of these gene editing strategies (comprehensively reviewed in [31]), the result is the precise introduction of a DSB.

**Fig. 2:**
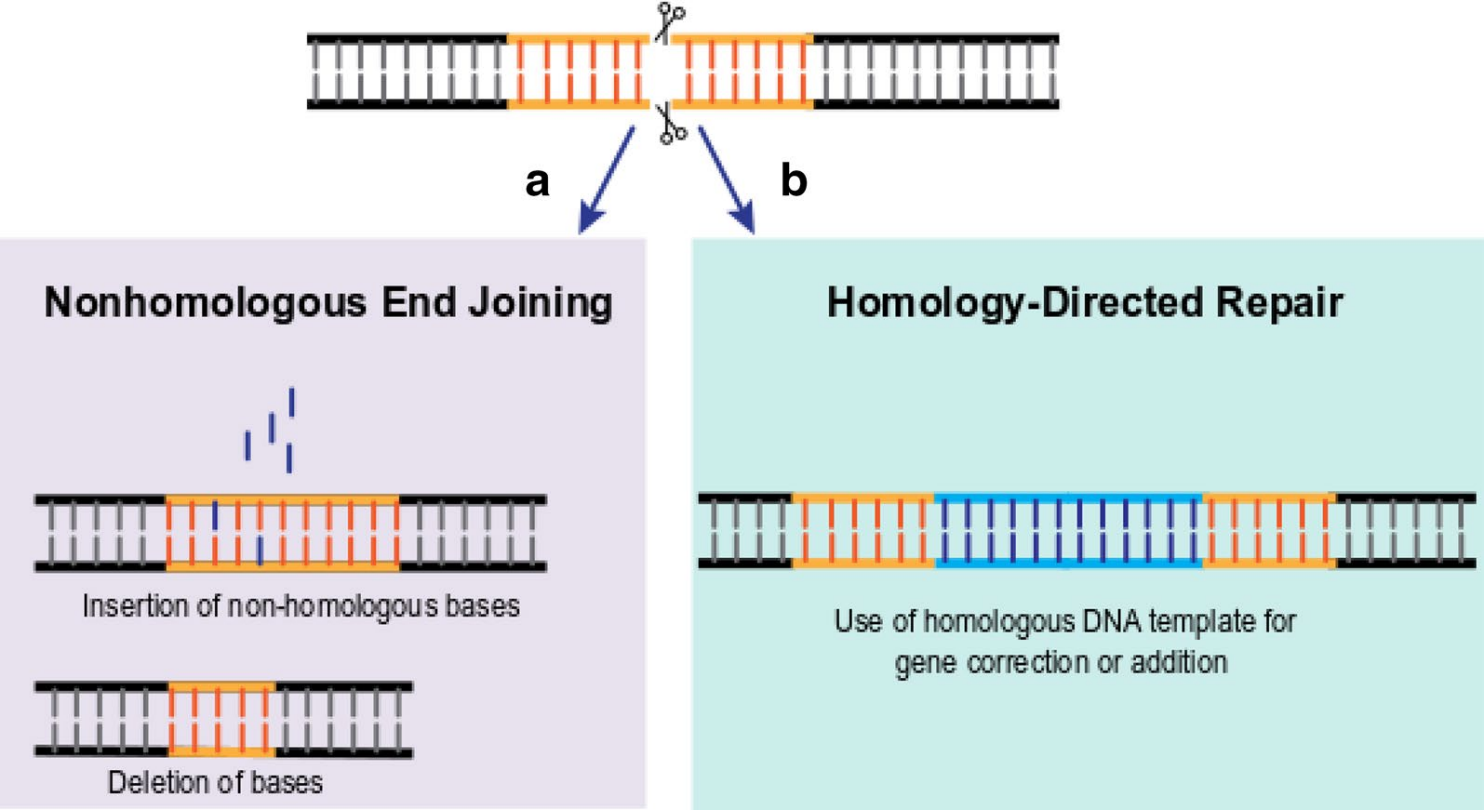
Mechanisms of double strand break repair exploited for gene editing. **a** Illustration of the results of the error-prone repair process during non-homologous end joining (NHEJ), which can introduce a mutation at the site of the double strand break through either the incorporation of random non-complementary nucleotides, or the deletion of nucleotides (indels). The goal is to either render a protein non-functional (e.g. knockout of diseased protein or preferentially knockout a functional protein for therapeutic benefit) or to (re-)activate a gene by either correcting/eliminating a deleterious nucleotide in the region of the break site or knocking out a repressive/inactivating element due to the introduction of an indel within that element. **b** Depiction of the results of homology-directed repair. Here a double strand break is induced in the presence of donor DNA. The donor DNA has nucleotide sequences flanking the gene to be inserted that are homologous to those upstream and downstream of the site of the break, enabling addition of the gene based on complementarity during the repair process

Following DSB repair is required. The predominant DNA-repair pathway for DSBs in mammalian cells is nonhomologous end-joining (NHEJ) [32]. NHEJ involves direct ligation of the DSB ends with negligible homology and thus is a highly error-prone process. It frequently leads to insertion or deletion of nucleotides (indels) in the DSB region, which can produce truncated proteins (Fig. 2a). This error-prone repair process is exploited in gene editing strategies to enable selective inactivation of genes (termed gene disruption) either to render pathogenic proteins non-functional or preferentially knock-out genes. NHEJ has already been exploited to aid cell therapy development. For example, gene-editing strategies capitalising on NHEJ-induced indels have been adopted to reactivate expression of foetal haemoglobin to compensate for faulty adult haemoglobin in distinct blood disorders [33].

DSBs can also undergo homology directed repair (HDR), a process primarily active during the S/G2 phase of the cell cycle, when homologous DNA is present in the nucleus. HDR can be utilised to selectively repair a deleterious mutation (targeted gene repair) or incorporate transgenes of interest within desired loci (gene addition) but it requires donor DNA to be co-delivered with the editing agent initiating the DSB, and for this DNA to remain available until cells reach S/G2, to enable incorporation during the repair process (Fig. 2b). HDR-mediated targeted repair is not a common approach in cell therapy, as mutations yielding pathogenic phenotypes can vary significantly between patients, necessitating a range of repair approaches. HDR is more commonly considered for targeted addition of transgenes, offering limited chromosomal positional effects and uniform transgene expression compared to virally mediated genetic engineering strategies. The resultant phenotype is predictable with limited risk of insertional mutagenesis making it an ideal choice for compensatory gene expression. For example, an in vivo gene editing strategy in a phase I clinical gene therapy trial for haemophilia B patients involves intravenous delivery of a ZFN that targets the albumin locus in hepatocytes (NCT02695160) [34]. The goal is to insert a healthy copy of the Factor IX gene, aberrant in these patients, into this locus to enable lifelong therapeutic production of the Factor IX clotting factor. Such in vivo gene therapies exploiting gene-editing strategies remain rare with only a handful approved globally (NCT02695160 for haemophilia B using ZFN by Sangamo; NCT02702115/mucopolysaccharidosis type I/ZFN/Sangamo; NCT03041324/mucopolysaccharidosis type II/ZFN/Sangamo). As the latter approaches are technically not considered cell therapies (cf. definition above) further discussion of these approaches is beyond the scope of this review. However, targeted gene addition in the context of cell therapies will be discussed below.

## Clinical trials using zinc finger nucleases

The first clinical use of gene edited cell therapies began roughly a decade ago. The first-in-human application of a targeted gene editing cell therapy involved disruption of the HIV co-receptor gene *CCR5* using zinc finger nucleases (ZFNs) in autologous CD4^+^ T-cells of HIV patients (NCT00842634) [35]. CCR5 is a transmembrane chemokine receptor expressed on the surface of activated T-cells, and is the major co-receptor for HIV-1 entry [36]. Following discovery of a homozygous 32-nucleotide deletion (Δ32) in a CCR5 allele which yielded a truncated protein not expressed on the cell surface in individuals with natural resistance to HIV-1 infection [37–39], early clinical trials investigated the potential for inhibiting HIV entry through CCR5 by blocking the HIV-CCR5 interaction using small molecule approaches [40]. Whilst these strategies showed some promise they would eventually result in viral selection for resistant mutants which were able to maintain CCR5 use for viral entry [41]. Given these strategies selected for resistance, a CCR5 knockout approach, analogous to the Δ32 genotype in endogenously resistant individuals, was considered optimal. CD4^+^ T-cells play a critical role in immune protection, and low counts in HIV-1 patients were strongly associated with progression to acquired immune deficiency syndrome (AIDS), which made this disease an ideal target for attempting treatment using a gene edited cell therapy [42]. Preclinical studies had identified a ZFN pair capable of producing a DSB within a transmembrane domain upstream of the naturally occurring Δ32 mutation in primary human CD4^+^ T cells. This ZFN pair resulted in a broad range of indels, but a specific five nucleotide addition (duplication of the sequence between ZFN binding sites) accounted for 30% of all sequence modifications, introducing two in-frame stop codons that prevented expression [43]. Following these promising pre-clinical results, in 2009 Sangamo Therapeutics initiated the first clinical trial to evaluate therapeutic safety (NCT00842634). Using an adenoviral vector delivery system, they reinfused autologous CD4^+^ T-cells edited ex vivo using their ZFN, named SB-728, into 12 patients. Results showed that the edited T-cells were safe in patients, with only one serious adverse event, attributed to a transfusion reaction, recorded. Additionally, some partial acquired resistance was reported where detectable HIV DNA levels decreased in most patients [35] pointing towards efficacy in this clinical trial. The use of an adenoviral approach allows high delivery efficiency but only transient ZFN expression, avoiding the complications of insertional mutagenesis experienced from earlier retroviral or lentiviral approaches.

The demonstration of clinical safety using this ZFN approach paved the way for future trials, with disruption of *CCR5* in cells from HIV patients currently being the most advanced clinical genome editing system. There are eight ongoing or completed clinical trials to date (Table 1). Subsequent clinical trials involved variations of parameters aimed at improving efficacy and homing in on patient populations most likely to benefit from different treatment regimens. Variations included modifying the input cell dose (NCT01044654), infusing multiple doses of edited cells (NCT02225665) and patient lymphodepletion by cyclophosphamide treatment prior to infusion of ZFN-edited T-cells, with the goal of enabling transient reduction of unedited T cell numbers to improve infused T-cell engraftment (NCT01543152). Scientists also moved to an mRNA electroporation method for ZFN delivery into T-cells (NCT02388594), as this strategy offered several safety advantages over DNA fragments derived from viral vectors, and a lower risk of insertion/mutagenesis at off-target sites as a result of shorter mRNA half-life compared to DNA [12]. These *CCR5* disruption approaches offer the potential for a ‘functional cure’ for patients if sufficiently high engraftment levels are achieved.

**Table 1:** Ongoing and completed clinical trials involving ZFN editing of the cell therapy. Based on data from https://clinicaltrials.gov/, 17th January 2020

Disease	Trial name	Phase	Cell type edited	Delivery and editing agent	Status	Sponsor	CT number
HIV	Autologous T-cells genetically modified at the CCR5 gene by zinc finger nucleases SB-728 for HIV (zinc-finger)	I	Autologous CD4^+^ T-cells	Adenoviral vector delivery, SB-728	Study completed—January 2013 (first posted—February 12, 2009)	University of Pennsylvania and Sangamo Therapeutics	NCT00842634
	Study of autologous T-cells genetically modified at the CCR5 gene by zinc finger nucleases in HIV-infected subjects	I/II	Autologous CD4^+^ T-cells	Adenoviral vector delivery, SB-728	Study completed—May 2015 (first posted—December 3, 2010)	Sangamo Therapeutics	NCT01252641
	Phase 1 dose escalation study of autologous T-cells genetically modified at the CCR5 gene by zinc finger nucleases in HIV-infected patients	I	Autologous CD4^+^ T-cells	Adenoviral vector delivery, SB-728	Study completed—December 2014 (first posted—January 8, 2010)	Sangamo Therapeutics	NCT01044654
	Dose escalation study of cyclophosphamide in HIV-infected subjects on HAART receiving SB-728-T	I/II	Autologous CD4^+^ T-cells	Adenoviral vector delivery, SB-728	Study completed—July 2017 (first posted March 2, 2012)	Sangamo Therapeutics	NCT01543152
	Repeat doses of SB-728mR-T after cyclophosphamide conditioning in HIV-infected subjects on HAART	I/II	Autologous CD4^+^ T-cells	Electroporated SB-728 mRNA	Study completed—June 2018 (first posted August 24, 2014)	Sangamo Therapeutics	NCT02225665
	A phase I study of T-cells genetically modified at the CCR5 gene by zinc finger nucleases SB-728mR in HIV-infected patients	I	Autologous CD4^+^ T-cells	Electroporated SB-728 mRNA	Study completed—May 2019 (first posted—March 17 2015)	University of Pennsylvania	NCT02388594
	Safety study of zinc finger nuclease CCR5-modified hematopoietic stem/progenitor cells in HIV-1 infected patients	I	CD34^+^ haematopoietic stem/progenitor cells	Electroporated SB-728 mRNA	Active, not recruiting—posted July 17, 2015; updated May 1, 2019	City of Hope Medical Center, Sangamo Therapeutic, California Institute for Regenerative Medicine (CIRM)	NCT02500849
	CCR5-modified CD4^+^ T cells for HIV infection (TRAILBLAZER)—T-cell reinfusion after interfering with lymphocyte binding location of AIDS virus through zinc-finger-nuclease elimination of CCR5 receptors	I/II	Autologous CD4^+^ T-cells	Adenoviral vector delivery, SB-728	Recruiting—posted September 12, 2018; updated July 24, 2019	Case Western Reserve University	NCT03666871
	A pilot study of T cells genetically modified by zinc finger nucleases SB-728mR and CD4 chimeric antigen receptor in HIV-infected subjects	I	Autologous CD4^+^ T- cells	Electroporated SB-728 mRNA	Recruiting—posted August 6, 2018; updated August 2, 2019	University of Pennsylvania	NCT03617198
	Long-term follow-up of HIV subjects exposed to SB-728-T or SB-728mR-T	I	Long-term follow-up of HIV-infected subjects who previously received SB-728-T or SB-728mR-T and completed 3 years of post-infusion follow-up		Enrolling by invitation, posted December 17, 2019; updated December 20, 2019	Sangamo Therapeutics	NCT04201782
Transfusion-dependent β-thalassemia	A study to assess the safety, tolerability, and efficacy of ST-400 for treatment of transfusion-dependent beta-thalassemia (TDT)	I/II	Autologous CD34^+^ haematopoietic stem/progenitor cells	Electroporated BIVV003/ST-400 mRNA	Recruiting—posted February 14, 2018; updated September 13, 2019	Sangamo Therapeutics and Bioverativ Therapeutics Inc.	NCT03432364
Sickle cell disease	A study to assess the safety, tolerability, and efficacy of BIVV003 for autologous hematopoietic stem cell transplantation in patients with severe sickle cell disease (PRECIZN-1)	I/II	Autologous CD34^+^ haematopoietic stem/progenitor cells	Electroporated BIV003/ST-400 mRNA	Recruiting—posted August 31, 2018; updated January 13, 2020	Bioverativ Therapeutics Inc.	NCT03653247

An alternative strategy using ZFN-edited haematopoietic stem/progenitor cells (HSPCs) has also been trialled, which was inspired by the results of the so-called “Berlin patient”. This HIV-1 infected patient, who now has an undetectable viral load [44, 45], underwent allogeneic CD34^+^ HSPC transplantation for acute myeloid leukaemia (AML) with HLA-matched cells from a donor homozygous for the Δ32 CCR5 allele [46]. Following this approach, preclinical studies established that ZFNs could be used to disrupt CCR5 expression in CD34^+^ HSPCs, which would also yield CCR5-negative differentiated progeny [47]. A further study established editing by ZFN mRNA electroporation offered less cytotoxicity compared to adenoviral vectors and was better scalable to levels required for clinical translation [48]. A follow-up trial implementing these aspects (NCT02500849) aimed to assess safety and feasibility of transplanting autologous CD34^+^ CCR5-negative HPSCs in patients who already have undetectable HIV viral loads from combination anti-retroviral therapy but suboptimal CD4^+^ cell levels. The goal was to provide patients with a HIV-resistant immune system without the need for an allogeneic donor, as progeny of the transplanted CCR5-negative HPSCs would also inherit the resistance. Here, the use of autologous cells was a huge advantage as one of the major risks for the strategy adopted in the Berlin patient was the possibility of graft-versus-host disease (GvHD). The pre-conditioning approach in this trial also differed to the total body irradiation approach of the Berlin patient, with trial patients receiving only the chemotherapeutic busulfan prior to edited HSPC infusions. This was a similar strategy as previously used in the second reported patient, the so-called “London patient”, which had an undetectable HIV viral load as a result of receiving an allogeneic CD34^+^ Δ32 homozygous HSPC transplant for blood cancer (Hodgkin’s lymphoma) [49, 50]. The London patient received a reduced intensity conditioning regimen exclusively of chemotherapy agents with known activity against lymphoma indicating potential success for this gene edited cell therapy approach. Peer-reviewed results from this follow-up clinical trial are still outstanding and whilst no longer recruiting, the trial is still active as follow-up assessments were scheduled for up to 5 years with completion expected in 2022. Results will be followed with interest by both the HIV and cell therapy communities.

For almost a decade Sangamo’s SB-728 ZFN for HIV patients was the only ZFN gene edited cell therapy entering clinical trials. However, when rival TALEN and CRISPR technologies emerged in clinical trials, Sangamo made the lateral move into blood disorders and pursued the potential for ZFN therapies for haemoglobinopathies, in collaboration with Bioverativ Therapeutics [51]. Haemoglobinopathy patients typically have mutations in the β-globin gene and thus produce malformed haemoglobin, such as in transfusion-dependent β-thalassemia (TDT) or sickle cell disease (SCD). Sangamo has developed ZFNs targeting the *BCL11A* gene, which is ordinarily involved in repressing production of foetal haemoglobin (Hbf) in adults [52, 53]. Hbf expression allows foetal haemoglobin to compensate for the malformed adult haemoglobin. By using a gene disruption approach, as seen with their CCR5 platform, the goal was to introduce indels that abrogate expression of the erythroid-specific enhancer of *BCL11A* in autologous CD34^+^ HSPCs [33, 54]. Following reinfusion and engraftment into patients this will enable mature adult blood cell progeny of the gene edited HSPCs to express high levels of endogenous HbF. The overall aim is to elevate HbF levels to the point where transfusion requirements for blood disorders such as TDT and SCD are alleviated. Whilst the first trials for TDT with the ZFN product ST-400 (NCT03432364) and SCD with BIV003 (NCT03653247) are still in the early stages of phase I, Sangamo has announced early results from the first patient treated in the TDT trial [55]. This patient has *β*^0^/*β*^0^ TDT, considered the most severe form [56], and neutrophil and platelet recovery at 2- and 4-weeks post gene edited HSPC infusion was demonstrated, respectively; this indicated successful reconstitution of haematopoiesis in this patient. By 7 weeks, HbF levels have risen from 1% of total haemoglobin to 31% suggesting successful gene editing by ST-400, supported by indels detected in peripheral blood. This patient previously received packed red blood cell transfusions every other week for the past 2 years and following ST-400 infusion continued to receive them for approximately 2 weeks, but subsequently they were no longer required (for at least another 5 weeks at the time of writing the corresponding publication [55]). Data from additional patients is expected toward the end of 2019, but these early results are encouraging and indicate that ZFNs still have a role to play in the growing cell therapy field.

The early clinical applications of ZFN cell therapy were focussed on HIV patients with many phase I/II trials now completed (Table 1). Despite the completed studies, but perhaps unsurprisingly given the cost and time required to collect, edit and expand sufficient numbers of T-cells/HSPCs for each patient, there is currently little activity to progress ZFN-based therapies toward phase III whilst long-term follow-up data continues to be gathered.

## Clinical trials with TALEN technology

Meanwhile, the first TALEN-edited cell therapies entering clinical trials have looked to overcome some of the challenges associated with ZFNs by adopting an ‘off-the-shelf’ approach (Table 2). CD19 is a transmembrane protein expressed on B-cells and B-cell precursors but not on bone marrow stem cells or other tissues. In blood cancers, it can be activated without extracellular stimulation required to transduce a signal, resulting in chronic B-cell activation. A number of promising genetic engineering strategies for autologous T-cell immunotherapy have been aimed at producing anti-CD19 CAR-Ts across a range of cancers to enable targeted treatment of these malignancies [57–59]; this includes commercially available CAR-T therapeutics such as tisagenlecleucel (Kymriah™) and axicabtagene ciloleucel (Yescarta™) which obtained approval in several countries [60, 61]. However, the number of patients able to benefit from the latter therapies are limited by the availability of high numbers of functional, expandable T-cells available from each individual patient. Moreover, the source T-cells could also be affected by the patients’ pre-treatment regimes. Notably, recent estimates suggest autologous CAR-T therapies cost around $95 k per dose to manufacture, with their allogeneic equivalents costing only around $4.5 k [62].

**Table 2:** TALEN cell therapy clinical trials. Based on data from https://clinicaltrials.gov/ last accessed 22nd January 2020

Disease	Trial name	Phase	Cell type edited	Target patients	Status	Sponsor	Countries	CT number
Cancer	Study of UCART19 in pediatric patients with relapsed/refractory B acute lymphoblastic leukemia (PALL)	I	Allogeneic T-cells	Patients with relapsed or refractory CD19-positive B-cell acute lymphoblastic leukaemia (B-ALL)	Recruiting—posted June 21, 2016; updated October 25, 2019	Institut de recherches internationales servier	USA, UK, Belgium, France, Spain	NCT02808442
	Dose escalation study of UCART19 in adult patients with relapsed/refractory B-cell acute lymphoblastic leukaemia (CALM)	I	Allogeneic T-cells	Patients with relapsed or refractory CD19-positive B-cell acute lymphoblastic leukaemia (B-ALL)	Recruiting—posted April 21, 2016; updated October 25, 2019	Institut de Recherches Internationales Servier	USA, UK, France, Japan	NCT02746952
	A study to evaluate the long-term safety of patients with advanced lymphoid malignancies who have been previously administered with UCART19/ALLO-501	I	Allogeneic T-cells	Patients with advanced lymphoid malignancies dosed with UCART19/ALLO-501 (long-term safety evaluation)	Enrolling by invitation—posted April 12, 2016; updated January 7, 2020	Institut de Recherches Internationales Servier	USA, Belgium, France, Spain, UK	NCT02735083
	Safety and efficacy of ALLO-501 anti-CD19 allogeneic CAR T cells in adults with relapsed/refractory large B cell or follicular lymphoma (ALPHA)	I/II	Allogeneic T-cells	Relapsed or refractory CD19 positive large B-cell lymphoma or follicular lymphoma patients	Recruiting—posted May 6, 2019; updated January 13, 2020	Allogene Therapeutics	USA	NCT03939026
	Study evaluating safety and efficacy of UCART123 in patients with acute myeloid leukemia (AMELI-01)	I	Allogeneic T-cells	Patients with CD123 expressing relapsed/refractory AML patients, and in poor-prognosis, newly diagnosed AML patients in the European LeukemiaNet (ELN) adverse genetic risk group	Recruiting—posted June 16, 2017; updated December 6, 2019	Cellectis S.A.	USA	NCT03190278
	Safety and efficacy of ALLO-715 BCMA allogenic CAR T cells in in adults with relapsed or refractory multiple myeloma (UNIVERSAL) (UNIVERSAL)	I	Allogeneic T-cells	Patients with relapsed or refractory multiple myeloma (MM) refractory to at least three prior lines of MM therapy	Recruting—posted September 18, 2019; updated December 12, 2019	Allogene Therapeutics	USA	NCT04093596
	Phase I study of UCART22 in patients with relapsed or refractory CD22+ B-cell acute lymphoblastic leukemia (BALLI-01)	I	Allogeneic T-cells	Patients with relapsed or refractory CD22+ B-cell acute lymphoblastic leukaemia (B-ALL)	Recruiting—posted November 4, 2019	Cellectis S.A	USA	NCT04150497
	Study evaluating safety and efficacy of UCART targeting CS1 in patients with relapsed/refractory multiple myeloma (MELANI-01)	I	Allogeneic T-cells	Patients with relapsed or refractory multiple myeloma (MM)	Recruiting—posted October 29, 2019; updated November 27, 2019	Cellectis S.A	USA	NCT04142619

Cellectis SA has developed an allogeneic approach termed Universal Chimeric Antigen Receptor (CAR) T-cells targeting CD19 (UCART19). It is intended to overcome limitations of the autologous approach and aims to offer a standardised therapeutic, with greater consistency, improved quality control and immediate availability to patients. The immunotherapy approach for tumour associated antigen (TAA) targeting follows a similar genetic engineering approach to autologous strategies already in development. Through lentiviral transduction, expression of a single chain variable fragment (scFv) targeting CD19 and linked to CD137 (4-1BB) and CD3ζ co-stimulatory/signalling domains is achieved in GMP grade. Importantly, the starting material are healthy donor peripheral blood mononuclear cells (PBMCs) [63, 64]. Additionally, UCART19 offers an enhanced safety profile over earlier therapies as the anti-CD19 scFv-41BB-CD3ζ is linked to an epitope marker/suicide gene (RQR8) that encodes target epitopes from CD34 and CD20, thereby allowing purification of the engineered population via CliniMACS^®^ CD34 selection. RQR8 is also a suicide gene, offering the option for binding of engineered CAR-Ts to the therapeutic monoclonal antibody rituximab which, in the event of unacceptable levels of toxicity in vivo, would allow selective destruction of transplanted cells [65]. The genetic engineering strategy to enable CD19 TAA targeting is coupled with a multiplexed gene editing approach to prevent GvHD and allow administration of UCART19 in non-HLA matched patients. Therefore, T-cells are electroporated with two pairs of TALEN mRNA targeting the TCRα constant gene (TRAC) and the CD52 gene locus. TRAC knockout yields TCR-negative cells as TCRαβ expression is dependent on formation of an αβ heterodimer, this mitigates the potential of alloreactivity with host cells, and any remaining TCR expressing cells can be depleted using CliniMACS^®^ TCRαβ after ex vivo expansion. CD52 knockout enables UCART19s to survive administration of the anti-CD52 mAb alemtuzumab, which is widely used as a lymphodepleting agent to prevent rejection of transplanted HLA mismatched cells [66] and as a conditioning therapy before therapeutic stem cell transplantation (SCT). In their first clinical application this multiplex strategy yielded a T-cell product with < 1% TCR expression with 85% of those cells expressing the CAR and 64% CD52-negative [64].

The preliminary results reported for the phase I paediatric acute B-cell lymphoblastic leukaemia (ALL) trial (PALL;NCT02808442) are promising for this difficult to treat patient group, with 5/5 patients achieving complete remission by day 28–42 after UCART19 infusion, and proceeding to conditioned allo-SCT 7 to 9 weeks later [67]. The corresponding dose escalation trial in adult patients (CALM; NCT02746952) reported 4/6 patients with complete remission (with incomplete blood count recovery, CRi) with no signs of minimal residual disease 28 days post-infusion (as determined by a tumour burden < 0.01% of cells assessed by flow cytometry and/or qPCR) [68]. Further pooled results for the CALM and PALL trials were reported in 2018, these suggest a manageable safety profile for UCART19 in the patients enrolled so far [69]. Whilst showing promising complete remission or CRi rates of 88%, the pooled results have also raised questions on how clinical status, tumour burden and pre-conditioning lymphodepletion impacts UCART19 expansion. These preliminary results suggest anti-leukaemic activity is connected to CAR expansion, as 2/16 patients evaluable for anti-leukaemic activity showed no UCART19 expansion and had refractory disease. Identical to UCART19 in molecular design, another Cellectis approach, ALLO-501, utilises a different manufacturing process with a different contract manufacturing organisation (CMO). Following the promising preliminary results of UCART19 Cellectis have licenced the rights of ALLO-501 to Allogene; and the first ALLO-501 clinical trial, in patients with large B-Cell or follicular non-hodgkin’s lymphoma opened for recruitment in the USA in May 2019 (NCT03939026). This selected target patient population has previously shown response to other anti-CD19 therapies [70].

Cellectis’ UCART123 was the second TALEN-edited cell therapy to enter clinical trials in 2017. UCART123 targets CD123 (interleukin (IL)-3 receptor α-chain), the primary low-affinity subunit of the IL-3 receptor, which is highly overexpressed in some haematological cancers. Here, Cellectis demonstrated TAA targeting by engineering expression of an anti-CD123 scFv linked to 4-1BB and CD3ζ domains, with the RQR8 epitope marker/suicide gene also incorporated. The same patented TALEN technology as in UCART19 was used to knockout TRAC to enable therapeutic use allogeneically [71, 72]. The phase I UCART123 trial currently underway for AML (NCT03190278) was approved in July 2019, with the first patient dosed in January 2020. This trial utilises a new UCART123 targeting construct and an optimised production process compared to the original investigational new drug (IND) status approved by the FDA [73]. This trial replaces Cellectis’ first AML UCART123 clinical trial which was put on hold by the FDA following adverse events in the first patients dosed due to cytokine release syndrome (CRS) [74]. Following an amendment lowering UCART123 maximum dosing, the trial resumed and an increased dosing regime was subsequently re-approved to accelerate development [75, 76], but no results were reported. Cellectis had previously registered two other UCART123 trials under their earlier UCART123 IND, another to treat AML patients in the UK (NCT04106076, acting as a sister trial to the previous USA AML trial) which was terminated in December 2019, as well as a trial for blastic plasmacytoid dendritic cell neoplasm (BPDCN) patients (NCT03203369) which was terminated at the end of July 2019. Study cessation in both instances was reported as a result of a sponsor decision, not a consequence of safety concerns. There was speculation this was due to low recruitment numbers (with zero and one participant recruited for each trial respectively at time of trial termination) or because of competition with Stemline Therapeutics Inc’s tagraxofusp, but Cellectis did not comment publicly on these decisions. Tagraxofusp (Elzonris™, SL-401), is a CD123-directed cytotoxin therapy and was the first drug and first CD123-targeted therapy approved for patients with BPDCN, demonstrating the potential for success of other CD123-targeted immune cell therapies such as UCART123.

Several other TALEN-edited allogeneic UCART products for haematological cancers have also recently entered phase I clinical trials (Table 2). In December 2019 the first B-ALL patient received the CD22-targeted UCART22 product (NCT04150497) [77]. CD22 is expressed in > 90% B-lineage cells in ALL and has a similar expression pattern to CD19 [78, 79], making anti-CD22 therapies suitable for use in event of resistance developing in patients receiving anti-CD19 therapies or for use as a combination therapy. Similar to the structure UCART19, UCART22 consists of scFv targeting the CD22 antigen, linked to 4-1BB and CD3ζ domains with TALEN-mediated TRAC and CD52 knock-out [80]. Two other TALEN edited UCART products targeting multiple myeloma (MM) antigens have also begun phase I trials—Cellectis’ UCARTCS1 product, targeting the CS1 antigen (NCT04142619) and the B-cell maturation antigen (BCMA)-targeted ALLO-715 product, licensed by Cellectis to Allogene Therapeutics (NCT04093596). As with Cellectis’ earlier UCART products UCARTCS1 and ALLO-751 also utilise multiplex engineering approaches, with mRNA delivered TALENs to knockout TRAC, minimising GvHD and expression of the CAR TAA targeting cassettes following lentiviral transduction incorporating the RQR8 suicide gene [81]. UCARTCS1 further utilises TALEN editing to knock-out CS1 from T-cells prior to CAR-cassette expression, to prevent cross-reactivity with UCART endogenous CS1 expression, as has been demonstrated in other T-cells engineered for CS1-targeting [82–84]. Both BCMA and CS1 (also known as SLAMF7/CD319) are highly expressed in MMs, with BCMA expressed in all MM cells in virtually all patients [85] whilst CS1 is expressed in > 95% of MMs [86]. Antibody immunotherapy trials targeting both these MM antigens (GSK2857916 and elotuzumab respectively) have shown good safety and tolerability [87–89], with elotuzumab remission rates of around 80% when administered in combination with lenalidomide and dexamethasone [90, 91], suggesting these new UCART products have promise.

Overall, like ZFNs, TALEN therapies are still in early clinical development, but confidence in design and manufacturing has developed rapidly, leading to four products entering phase I trials in the last quarter of 2019 (Table 2). Cellectis and their licenced partners so far have a monopoly on this editing modality and have focused exclusively on immuno-oncology using well-established target antigens that have shown promise in autologous and antibody-mediated immunotherapies. Combined with the fact that the most established of these studies are already operating in centres across multiple countries it would not be surprising to quickly see TALEN-edited cell therapies surpass the milestones of their ZFN predecessor.

## Clinical trials using CRISPR technology

Whilst the CRISPR gene editing system was discovered about 2 years after TALENs, it followed the first TALEN cell therapy into clinical trial just a year later. As with the TALEN therapy UCART19 the gene edited cells used in the first CRISPR trial were also T-cells, but in this instance they were used to treat a patient with advanced non-small cell lung cancer, as part of a phase I trial (NCT02793856) [92]. Like many subsequently registered CRISPR cell therapy trials the focus was on the use of autologous T-cells, edited using CRISPR–Cas9 to knock out the immune checkpoint inhibitor programmed cell death-1 (PD1) prior to reinfusion back into the patient. PD-1 is an inhibitory TCR transiently up-regulated during early activation of T-cells and ordinarily involved in the regulation of immune tolerance, acting to decrease autoimmune reactions, however this inhibitory action also enables cancers to evade immune mediated elimination. Additionally, PD-1 has been recognised as a marker of T-cell exhaustion, as a consequence of perpetual T-cell stimulation, often resulting in tumour re-emergence [93]. Neutralising antibodies for PD-1 or its ligand programmed cell death ligand 1 (PD-L1) have already shown promise as an immunotherapy for a range of cancers, including non-small cell lung cancer [94–99], heralding its potential as a target for gene-knockout. Yet, it still remains to be seen if knockout engineered T-cells offer significant enough clinical benefits over PD-1 or PD-L1 antibodies, when considering the laborious and costly process of genetic modification and T-cell propagation. This first-in man phase I study has already preliminarily reported on nine patients and is no longer actively recruiting participants [100, 101]. Split into three cohorts, participants received either 1 × 10^7^/kg, 2 × 10^7^/kg or 4 × 10^7^/kg edited T-cells with the later cohorts receiving the escalated doses. The study reported no serious adverse events at any of the doses, with two patients experiencing 17.6- and 22.0-weeks stable disease, with median progression-free survival of 7.6 weeks. The study concluded that the therapy was safe and larger studies are required to explore effective doses. The indicated safety is promising for other CRISPR trials underway, as several more are in the early stages for a range of different cancers (Table 3).

**Table 3:** Ongoing CRISPR cell therapy clinical trials. Based on data from https://clinicaltrials.gov/ last accessed 17th January 2020

Disease	Trial name	Phase	Cell type edited	Target patients	Status	Sponsor	Countries	CT number
Cancer	PD-1 knockout engineered T cells for advanced esophageal cancer	II	Autologous T-cells	Patients with recurrent or metastatic oesophageal cancer	Study completed—February 2018 (first posted: March 16, 2017)	Hangzhou Cancer Hospital	China	NCT03081715
	PD-1 knockout engineered T cells for metastatic non-small cell lung cancer	I	Autologous T-cells	Patients with stage IV non-small cell lung cancer with measurable lesions	Active, not recruiting—posted June 8, 2016; updated August 5, 2019	Sichuan University	China	NCT02793856
	PD-1 knockout EBV-CTLs for advanced stage Epstein–Barr virus (EBV) associated malignancies	I/II	Autologous T-cells	Patients with Epstein–Barr virus^+^ve stage IV malignancies including: gastric carcinoma, nasopharyngeal carcinoma, T-cell lymphoma, adult hodgkin lymphoma, diffuse large B-cell lymphoma	Recruiting—posted February 7, 2017; updated May 2, 2017	Yang Yang	China	NCT03044743
	NY-ESO-1-redirected CRISPR (TCRendo and PD1) edited T Cells (NYCE T Cells)	I	Autologous T-cells	Patients with relapsed refractory multiple myeloma (MM), melanoma, synovial sarcoma, or myxoid/round cell liposarcoma (MRCL)	Active, not recruiting—posted January 16, 2018; updated January 6, 2020	University of Pennsylvania	USA	NCT03399448
	Study of CRISPR–Cas9 mediated PD-1 and TCR gene-knocked out mesothelin-directed CAR-T cells in patients with mesothelin positive multiple solid tumors	I	Autologous T-cells	Patients with mesothelin positive tumours that have failed at least one standard care chemotherapy for advanced disease	Recruiting—posted June 4, 2018; updated December 18, 2019	Chinese PLA General Hospital	China	NCT03545815
	Study of PD-1 gene-knocked out mesothelin-directed CAR-T cells with the conditioning of PC in mesothelin positive multiple solid tumors	I	Autologous T-cells	Patients with mesothelin positive tumours that have failed ≥ 1 standard care chemotherapy for advanced disease, particularly: Pancreatic, cholangiocarcinoma and ovarian cancers	Recruiting—posted November 20, 2018; updated November 20, 2018	Chinese PLA General Hospital	China	NCT03747965
	A feasibility and safety study of universal dual specificity CD19 and CD20 or CD22 CAR-T Cell immunotherapy for relapsed or refractory leukemia and lymphoma	I/II	Allogeneic T-cells	Patients with relapsed or refractory CD19^+^ B-cell leukaemia or lymphoma	Recruiting—posted January 16, 2018; updated January 16, 2018	Chinese PLA General Hospital	China	NCT03398967
	A study evaluating UCART019 in patients with relapsed or refractory CD19^+^ leukemia and lymphoma	I/II	Allogeneic T-cells	Patients with relapsed or refractory CD19^+^ B-cell leukaemia or lymphoma	Recruiting—posted May 25, 2017; updated June 23, 2017	Chinese PLA General Hospital	China	NCT03166878
	A safety and efficacy study evaluating CTX110 in subjects with relapsed or refractory B-cell malignancies	I/II	Allogeneic T-cells	Patients with relapsed or refractory non-hodgkin’s lymphoma	Recruiting—posted July 29, 2019; updated December 10, 2019	CRISPR Therapeutics AG	USA, Australia	NCT04035434
	CRISPR (HPK1) edited CD19-specific CAR-T cells (XYF19 CAR-T cells) for CD19^+^ leukemia or lymphoma	I	Autologous T-cells	Patients with relapsed or refractory CD19^+^ B-ALL or other B-cell lymphomas	Recruiting—posted July 30, 2019; updated July 30, 2019	Xijing Hospital	China	NCT04037566
HIV	Safety of transplantation of CRISPR CCR5 modified CD34^+^ Cells in HIV-infected subjects with hematological malignancies	I/II	Autologous CD34^+^ HSPCs	Patients on cART with undetectable viral load and a haematological neoplasm	Recruiting—posted May 23, 2017; updated May 23, 2017	Affiliated Hospital to Academy of Military Medical Sciences	China	NCT03164135
Transfusion-dependent β-thalassemia	A safety and efficacy study evaluating CTX001 in subjects with transfusion-dependent β-thalassemia	I/II	Autologous CD34^+^ HSPCs	Homozygous β-thalassemia patients (excluding β0/β0 genotype) or compound heterozygotes including β-thalassemia/haemoglobin E (HbE)	Recruiting—posted August 31, 2018; updated December 5, 2019	Vertex Pharmaceuticals Incorporated	Canada, Germany, UK, USA	NCT03655678
Sickle cell disease	A safety and efficacy study evaluating CTX001 in Subjects with severe sickle cell disease	I/II	Autologous CD34^+^ HSPCs	Sickle cell patients with βS/βS genotype and ≥ 2 vaso-occlusive crisis events yearly for past 2 years	Recruiting—posted November 19, 2018; updated November 21, 2019	Vertex Pharmaceuticals Incorporated	USA, Germany, Italy, Belgium, Canada	NCT03745287

China is currently leading the way in CRISPR cancer cell therapy trial numbers, with their first competition in the form of the University of Pennsylvania-sponsored ‘NY-ESO-1-redirected CRISPR (TCRendo and PD1) Edited T Cells (NYCE T Cells)’ trial (NCT03399448) [102]. As with the TALEN trials a multiplex genome editing approach is used, combining lentiviral transduction to enable expression of a TCR specific for NY-ESO-1 with triple gene editing using Cas9-complexed guide RNAs targeted to disrupt expression of endogenous TCRα, TCRβ and PD-1. NY-ESO-1 is a highly immunogenic cancer/testis antigen expressed in a range of malignancies, but not ordinarily expressed in normal tissues, besides the placenta and testis [103, 104]. Spontaneous antibody and T-cell reactions are often reported in patients with advanced tumours expressing NY-ESO-1, and in advanced myelomas NY-ESO-1 expression is correlated with tumour proliferation [103–105]. Unsurprisingly, given its restricted re-expression profile, many immunotherapy trials targeting NY-ESO-1 are already underway, as comprehensively covered by Thomas et al. [106]. The NYCE multimodal trial, whilst one of the first CRISPR clinical trials, followed on from a phase I/II trial previously published that indicated use of transgenic T-cells with NY-ESO-1 targeting is safe, with cells capable of long-term engraftment (engineered cells detected in 9/10 patients who reached 2-year follow up) and homing to tumour sites, as well as retaining cytotoxic potential over time (for up to a year after infusion) [107]. By combining the previous strategy with endogenous TCR knockout the aim of incorporating the gene editing approaching with NY-ESO-1 targeting is to minimise TCR mispairing and competition of the transduced NY-ESO-1 TCR with endogenous TCRs and potential neo-reactivity or autoimmunity [108, 109], whilst PD-1 knockout is intended to preserve activity of T-cells by preventing exhaustion [110].

Besides genome editing approaches targeting PD-1 for knockout in immuno-oncology there is significant interest in the potential of CRISPR-edited cell therapies for the treatment of blood disorders. CTX001 is the first in this class to reach the clinical trial stage and was developed in partnership by CRISPR Therapeutics Inc. and Vertex Pharmaceuticals Inc. It is the first industry-sponsored CRISPR therapeutic and competes for the same patient populations as Sangamo’s ZFN products ST-400 and BIV003. It also uses a similar strategy, aiming to abrogate *BCL11A* expression in autologous CD34^+^ HSPCs, albeit using CRISPR–Cas9, prior to expansion of edited cells and infusion into the patient. Use of CTX001 in TDT patients was its first application (NCT03655678) about 6 months after the ST-400 trial began, with treatment of SCD patients (NCT03745287) following roughly 2.5 months after the BIV003 trial (see above). Preliminary safety and efficacy data is expected towards the end of 2019, but reports of the first patient treated in the TDT trial are encouraging given they remain transfusion-independent for longer than 4 months post engraftment [111].

CRISPR therapeutics have recently entered the crowded market of CD19-targeted CAR-Ts with the launch of their first CTX110 clinical trial (previously known as CTX101, NCT04035434). Utilising a multiplex genome editing approach in allogeneic T-cells they have inserted a CD19-targeted CAR into the TRAC locus, simultaneously eliminating TCR expression and yielding consistent CAR expression. Additionally, they have knocked out the β2-microglobulin (B2M) gene to eliminate MHC class I expression, with the aim of improving CAR-T durability. CTX110 represents the first gene-edited cell therapy with a targeted insertion to be reported entering the clinic, although it was originally intended to begin clinical trial in 2018. Despite the relative technical difficulty of high-efficiency gene insertion approaches CRISPR Therapeutics report consistent results of 54–66% cells yielding all three desired edits (B2M and TRAC knockouts and CAR expression from the TRAC locus) across five different donors, indicating such a strategy holds promise for their future pipeline [112].

Finally, in a throwback to the first gene-edited cell therapy clinical trial, there is also interest in CRISPR-editing of CCR5 in HIV patients, with results of the first patient to receive a bone marrow transplant of CCR5-KO CD34^+^ HPSCs recently published [113]. Interestingly this report is one of the few CRISPR platforms so far to detail the method of delivery for the editing agent, in this case a ribonucleoprotein complex comprising Cas9 protein and their previously designed gRNAs targeting CCR5 [114], while most other platforms use proprietary delivery methods. The results demonstrated an acceptable safety profile, with no immunogenicity and no detected off-target effects following analysis of whole-genome sequencing data for translocations or long-range deletions. Whilst this data would suggest that gene editing technologies have now gone full circle, potentially preparing to eliminate the need for ZFNs, these results are limited to a single case report for an individual patient and demonstrate relatively low HPSC editing efficiency (17.8% of cells) compared to their predecessor (54–67% [48]) demonstrating there is some way to go before this strategy can compete with more established methods.

## Off-target effects and preclinical developments in gene editing strategies

One factor that remains a fundamental concern with any gene edited cell therapy and becomes increasingly so as multiplex approaches are progressively adopted, is the potential for off-target effects as a result of nuclease activity at unintended homologous sites; and any downstream consequences arising from such off-target activity. As the range of cell therapies available to treat patients advances over time, so too will the range of targets for editing. Consequently, the ability to accurately identify potential off-target sites becomes increasingly important. Currently, a growing range of strategies to address off-target activity by assessing secondary target sites exist, including SELEX, Digenome-seq, GUIDE-seq, CIRCLE-seq and DISCOVER-seq [115–119]. Mutagenesis levels within cells at the identified sites are preferentially examined by these deep sequencing and targeted PCR approaches, as opposed to whole genome sequencing (WGS) which lacks adequate sequencing depth to detect low frequency mutations in bulk populations of cells. This was demonstrated in the early SCID-X1 retroviral-mediated gene therapy trials where *LMO2* proto-oncogene activation in hSCs was estimated to be present in between only 1 and 10 of the ≥ 1 × 10^6^ transduced cells transplanted to the patients who subsequently went on to develop leukaemia [15]. That said, WGS approaches are suitable in instances examining single cell-derived clones such as human induced pluripotent stem cells (hiPSCs), which can be used for clonal expansion prior to therapy, as is the case for upcoming iCART or iPSC-derived NK cells [120, 121] (for details see “Future perspectives of gene edited cell therapies in the clinic” section).

It is important to note that off-target concerns will vary depending on the cell therapy, with risks restricted to only a subset of the genome relevant to the cell type [122]. For example, in the discussed therapies for treating haemoglobinopathies concerns would be related to off-target effects that would impact cells of the haematopoietic lineage, so identifying possible secondary binding sites and assessing their potential impact in those cell types would provide the necessary reassurance of therapeutic safety. This would require screening for and rejecting strategies that present a risk of tumour suppressor gene mutations for instance, as this could result in leukaemia downstream. However off-target effects in a muscle-specific gene, such as dystrophin, which to our knowledge is neither expressed nor necessary in hematopoietic lineage cells may be considered tolerable. Conversely, mutations found in the β-globin gene may be considered tolerable in a muscular dystrophy cell therapy [122]. This is akin to the accepted risk–benefit analysis of side effects in the clinic, where drugs such as small-molecules and antibodies can interact with off-target, structurally similar proteins but the on-target efficacy is sufficiently beneficial for these risks to be deemed acceptable. Here, use of a cell therapy approach is beneficial, because gene editing is restricted only to known populations of ex vivo cultured cells, which can be easily screened, rather than the requirement to ensure successful delivery of the editing agent to specific tissues and more invasive procedures for downstream screening such as biopsies, as with in vivo gene editing [123].

Finally, improved in silico approaches to predict genome wide off-target activity are set to continue alongside the development of machine-learning methods which are benefitting from the increasing availability of large-scale genome-editing activity datasets [124–127]. Together with these advances in predicting potential off-target effects researchers are continually pursuing approaches to improve genomic targeting precision. For ZFNs this has included improving cleavage activity by reducing undesired homodimerization of the ZFN pairs through modification of FokI domains [128]. TALEN technologies have focused on virtually eliminating low frequency off-site effects by replacing naturally occurring repeat-variable di-residues (RVDs) within modular TALE arrays (which are responsible for specifying the target nucleotide for binding), using unconventional TALEs not present within the natural TALE repertoire [129–131]. These have significantly improved targeting specificity and offer a more simplified means to facilitate multiplex editing strategies for therapeutic use. In the CRISPR field a range of strategies have been tested to improve specificity, including decreasing the gRNA length with truncated gRNAs [132] and engineered Cas9 variants with reduced non-specific protein–DNA interactions such as structure-guided engineered Cas9s [133, 134]. Other strategies have sought to build on the success of the paired approaches used by their ZFN and TALEN predecessors by using Cas9-nickases (Cas9-n). These modified Cas9 enzymes have been mutated at the nuclease active site to yield single rather than DSBs and can be paired to generate composite DSBs offering double the genome editing specificity of traditional Cas9 approaches [135, 136]. A new approach building on Cas9-ns called ‘prime editing’ may in the future be of particular interest for multiplex strategies or targeted insertion/repair approaches. Prime editing utilises a reverse transcriptase enzyme fused to both an RNA-programmable Cas9-n and a prime editing guide RNA (pegRNA) [137]. This enables genetic information to be directly copied from the extending pegRNA into the target genomic locus without relying on DNA repair or exogenous donor templates, with the higher efficiency of this approach demonstrated by the 270-fold higher ratio of editing:indels seen when prime editing was evaluated relative to a comparable Cas9-initiated HDR strategy in 293T cells [137]. Prime editing is unlikely to be used to make the large insertions or deletions current gene editing/CRISPR approaches are implementing because the long RNA strands required would likely be enzymatically degraded within cells before editing could be achieved. The versatility of prime editing holds significant promise for a range of genetic diseases caused by targetable multi-base mutations such as sickle cell anaemia, Duchenne muscular dystrophy, cystic fibrosis and Tay–Sachs disease and further development of this technique will be followed with interest by the cell therapy community.

## Future perspectives of gene edited cell therapies in the clinic

Whilst gene editing in the context of cell therapies has mostly concentrated on immunooncology or blood disorders, recent advances of human induced pluripotent stem cell (hiPSC)-derived therapies to first-in-man studies indicate an increased breadth in application is forthcoming. hiPSCs, like embryonic stem cells (ESCs), have unlimited self-renewal potential and can differentiate into all adult cell types, but as they are derived from somatic cells, they can be generated from donors whose genetic characteristics and health records are well-established, without the ethical implications of ESCs. Several first-in-man studies using hiPSC-derived cells are already underway to treat patients with a variety of aetiologies including dopaminergic neurons for Parkinson’s disease [138–141], retinal pigment epithelial (RPE) cells and corneal cells for eye diseases [142–146], cardiac progenitors for heart failure ([147, 148] and NCT03763136) and mesenchymal stem cells for steroid resistant GvHD (NCT02923375 [149]). Many of these therapies have reached the clinic sooner than would have been anticipated as a result of recent legislative changes in Japan, which allowed stem cell treatments fast track approval to clinical use on the basis of safety and efficacy tests using only small patient numbers [150–152]. Nevertheless, wider clinical use of hiPSC-therapies could have been limited by the prohibitive costs and time associated with the development of autologous hiPSC lines. An autologous strategy was used for the first patient treated with a hiPSC-derived cell therapy, in this case RPE cells for macular degeneration [144], and this cost around $1 million, and took over 10 months to produce the cells for transplant [153]. Fortunately, all subsequent clinical use has relied on allogeneic hiPSCs, with the first allogeneic hiPSC-derived cell therapy also using RPE cells, following the same procedure as its autologous predecessor, but with time to surgery reduced to a month and the cost reduced to $200 k per patient [146, 153].

The majority of allogeneic hiPSC lines used have come from the Center for iPS Cell Research and Application at Kyoto University (CiRA). CiRA has been focused on setting up allogeneic hiPSC stocks by selecting rare donors that are homozygous at the three major HLA gene loci, to generate a pool of safe clinical grade hiPSC clones [153]. This would lead to reduced risk of immune rejection following transplantation of differentiated cells if the recipient HLA haplotypes are matched. Once completed the bank is estimated to offer coverage for up to 90% of the Japanese population, but at the cost of comprehensive safety and characterisation testing of as many as 140 lines, following screening of > 150,000 donors. Japan has a less genetically diverse population than the rest of the world, so whilst other banks including the UK based Cell & Gene Therapy Catapult and Cellular Dynamics Inc in the U.S. are also generating hiPSCs from HLA homozygous donors a large number of lines would need to be generated to approach the same level of coverage globally [154, 155].

A competing approach that shows great promise and may herald the next wave of gene-edited cell therapies could involve utilising hiPSC lines that have undergone gene editing to modify the HLA genes. HLA pseudo-homozygous hiPSC lines were recently generated through CRISPR–Cas9 editing of heterozygous HLA class I donor derived lines [156]. Using a precise allele-specific multiplex approach biallelic deletion of HLA-A and HLA-B genes, but retention of a single HLA-C allele was achieved. This approach offers a solution to hurdles in other HLA editing approaches which aimed to create ‘hypoimmunogenic’ hiPSCs by knock-out of the B2M gene [157]. Whilst hypoimmunogenic strategies would prevent immune rejection by depleting all HLA loci, it also precludes natural immune destruction of oncogenically transformed or infected cells post-transplant, as well as exposing these cells to potential destruction by natural killers cells as a result of their HLA-C deficiency [157, 158]. It is predicted that just 12 of these alternative ‘pseudo-homozygous HLA-C retained’ hiPSC lines would be immunologically compatible with > 90% of the global population, which would greatly enhance hiPSC-derived cell therapy applications. Given that CTX110, which involves B2M knockout by CRISPR–Cas9, has already entered phase I trials in the USA and Australia it seems possible regulators could accept additional HLA-edited cell therapies in the clinic in future.

While the debate on the best means to enable globally compatible immune coverage continues, new ‘off-the-shelf’ hiPSC cell therapies suitable for mass production are already starting to emerge in the immunooncology arena. US-based Fate Therapeutics’ (FT) first “Off-the-shelf” iPSC-derived NK cell products FT500 and FT516 are already in phase I clinical trials (NCT03841110, NCT04023071) and their first iPSC-derived CAR-T cell product FT819 looks set to be the first gene-edited hiPSC cell therapy to enter the clinic. It follows a similar strategy to autologous CD19 CAR-Ts and CTX110 in particular. FT819 is differentiated from a hiPSC clonal master line that has undergone CRISPR-mediated disruption of TCR expression by insertion of a novel 1XX CAR signalling domain into the TRAC locus in a similar 2-in-1 TCR-knockout/CAR-knock-in strategy, simultaneously mitigating GvHD risk whilst providing potent targeting [159, 160]. FT819 will compete with Takeda Pharmaceutical Company Ltd, who are in a 10-year partnership with CiRA to develop iPSCs clinically, to be the first hiPSC-derived CAR-T (iCART), as Takeda plan to start clinical trials with their first iCART products in 2021, a CD19-targeted CAR amongst those reported [161]. The real promise of gene edited hiPSC-derived cell therapies has yet to be fulfilled, but editing prior to differentiation into a wide range of cell therapies may ultimately make universal therapies more economically viable in the future, though there is clearly some work to be done to bring down the costs of differentiation protocols before this field matches pace with their edited primary cell counterparts.

## Conclusions

Thus far editing strategies entering clinical trial have predominantly focused on gene disruption, either to enable deletion of protein expression for therapeutic gain (e.g. PD-1, CCR5) or for targeted correction of disease phenotypes by compensatory gene reactivation (BCL11A) with many of the therapies on the horizon taking similar approaches. Whilst increasingly sophisticated multiplex approaches are also beginning to emerge, with CTX110 the first targeted gene insertion to enter trial, much more research is likely to be needed before more sophisticated safety features such as reporter genes or suicide cassettes become common place. UCART19 and UCART123 have likely already paved the way for regulatory bodies to accept strategies incorporating suicide genes, with their epitope marker/suicide gene RQR8 already employed as part of their multiplex engineering approach. Moreover, as reporter genes and in vivo imaging become increasingly employed to track cell fate in autologous cell therapy approaches [5, 9, 162–167], it is likely that future allogeneic and off-the-shelf gene edited therapies, particularly those derived from iPSCs, will consider incorporating these strategies to offer regulators reassuring safety data by longitudinal therapy tracking.

## Data Availability

Not applicable.
